# New Effective Method of Lactococcus Genome Editing Using Guide RNA-Directed Transposition

**DOI:** 10.3390/ijms232213978

**Published:** 2022-11-12

**Authors:** Pavel Yu Pechenov, Danil A. Garagulya, Daniil S. Stanovov, Andrey V. Letarov

**Affiliations:** Federal Research Center “Fundamentals of Biotechnology” of Russian Academy of Sciences, Leninsky Prospect, 33, Build. 2, 119071 Moscow, Russia

**Keywords:** *Lactococcus lactis*, genomic engineering, transposon-associated CRISPR–Cas system

## Abstract

*Lactococcus lactis* is an important industrial microorganism and a widely used model object for research in the field of lactic acid bacteria (LAB) biology. The development of new *L. lactis* and related LAB strains with improved properties, including phage-resistant strains for dairy fermentation, LAB-based vaccines or strains with altered genotypes for research purposes, are hindered by the lack of genome-editing tools that allow for the easy and straightforward incorporation of a significant amount of the novel genetic material, such as large genes or operons, into the chromosomes of these bacteria. We recently employed a suggested system based on the CRISPR–Cas-associated transposon for the editing of the *L. lactis* genome. After the in-depth redesign of the system, we were able to achieve the stable incorporation of the fragments that were sized up to 10 kbp into the *L. lactis* beta-galactosidase gene. The efficiency of editing under the optimized conditions were 2 × 10^−4^ and 4 × 10^−5^ for 1 kbp and 10 kbp, respectively, which are sufficient for fast and easy modifications if a positive selection marker can be used.

## 1. Introduction

*Lactococcus lactis* is one of the most important industrial microorganisms that is used in the dairy and food industries, in commodity chemicals production and in enzyme and metabolite biotechnological production [1,2,3]. This species has also been considered for medical applications such as the lactococcal vaccine vectors, including a candidate mucosal vaccine against malaria [4]. A relatively small genome size of about 2.3 Mbp makes *L. lactis* an attractive model object for lactic acid bacteria (LAB) biology research. Currently, the plasmid expression vectors and shuttle cloning vectors are available for the *L. lactis* system [5,6]. At the same time, the existing *Lactococcus* genome editing toolkit is very limited. The most commonly used approach for *L. lactis* chromosome engineering is based on RecA-mediated recombination with non-replicative or conditionally replicative plasmids [7,8,9]. In two such studies, the homologous recombination was used to integrate longer (up to 5 kbp) fragments [10,11], however, the output of these protocols is critically depending on the transformation efficacy. From the practical perspective, the low frequency of the RecA-mediated recombination makes it quite time-consuming for these methods to generate a prospective mutant (up to 3 weeks), and they are labor-intensive, even if the counter-selection markers are employed [12].

Recently the CRISPR/Cas-based methods for LAB genomes editing were developed [13,14]. The use of CRISPR–Cas9 in combination with the RecT-mediated recombination allowed researchers to perform *L. lactis* genome editing with 75% efficiency, however the authors were not able to obtain insertions of the fragments that were longer than 150 bp [12]. In another study, a single-plasmid CRISPR–Cas9-based system allowed the researchers to generate deletions of large (~1 kbp) genomic fragments but not insertions of the genetic material [14].

The development of more powerful genome-editing methods may provide novel opportunities for the investigation of the LAB biology, the re-programming of their metabolism, including the elimination of unwanted reactions and directing the metabolic pathways to produce the compounds of interest, as well as for developing new lactococcal probiotics and vaccines [15,16].

In 2018, the natural CRISPR–Cas-associated transposons were discovered [17,18], in which the complex of the Cas proteins and guide RNA directed the transposition of the mobile elements. These systems were employed to create an effective tool to produce long insertions in the chromosomes of many bacterial species [19,20].

The INTEGRATE (insert transposable elements by guide RNA-assisted targeting) system developed by Klompe et al. [21] employs the Cas678 effector complex from the type I-F CRISPR–Cas system from *Vibrio cholerae HE-45* and the genes of the transposase TnsABC-TniQ that acts in trans to catalyze the transposition of a cargo DNA that has been inserted between the transposase recognition sequences (transposon shoulders) in the donor plasmid. The transposition site is determined by the sequence of the guide RNA, which is associated with the Cas678 complex. The system allows research to integrate long genetic cassettes into the genomes of Gram-negative organisms such as *Escherichia coli*, *Klebsiella oxytoca* and *Pseudomonas putida*. Interestingly, in addition to the standard protospacer adjacent motive (5′- PAM) sequence CC, the 5′–PAM could be represented by the dinucleotides CA, GT, CA, GC, CG and CT, allowing the researchers more freedom to choose the integration site, though the efficiency with such alternative PAM sequences can be lower. The off-target activity of this system is below 5% which makes it a valuable instrument in bacterial genomes editing. However, in most of the cases, the direct use of the genetic systems from *E. coli* and other Gram-negative bacteria in lactococci is hindered by the differences in the promoters and ribosome binding sites sequences in these bacterial species.

Here, we developed a new tool for LAB genomes editing that is based on the Cas guide RNA-directed transposition system that has been described by Klompe et al. [22] and is adapted to the function of the *L. lactis* cells. This system allows for the rapid (1.5 weeks to obtain a desired mutant) and precise editing of the *L. lactis* genome, including the insertion of long DNA sequences.

## 2. Results

### 2.1. Development of the Genome-Editing System

At the initial point of our work, we used the triple-plasmid system based on the transposon-associated nuclease-deficient CRISPR/Cas element from *V. cholerae HE-45*, which had been suggested earlier for *E. coli* editing [22]. In this tool, the protein guide RNA complex catalyzing the transposition is expressed from two plasmids: pQCascade, encoding the Cas proteins and the guide RNA, and pTnsABC, providing the transposase subunits. The integrative sequence, represented by the promoter-less chloramphenicol resistance gene that was inserted between the shoulders of transposon, is provided by the third plasmid pDonor. For the editing, all three of the plasmids should be co-transformed into the strain of interest. The positive clones can be then selected by the antibiotic resistance which emerge once the marker-containing sequence is inserted into a locus that is transcribed from any cellular promoter (the requirement somewhat limits the choice of the integration sites). In order to adapt this system for the use in *L. lactis*, we re-designed it into a two-plasmid version that consists of the vectors, pL2INT and pLcDonor.

The pL2INT plasmid was created on the basis of the pTRKL2Sp vector which is able to replicate in *Lactococcus*. (It has to be noted that the pTRKL2Sp vector has the spectinomycin selective marker and after the transformation of *L. lactis* with this plasmid or its derivatives, one frequently observes two colony morphotypes: small and large. The small colonies most often do not contain the plasmid, and seemingly, they arise from spontaneous mutants with partial resistance to spectinomycin. The larger colonies, which are comparable in size to the colonies observed on the medium without the antibiotic, are the true transformants).

The pL2INT plasmid contains the operon encoding all of the proteins of the Cas complex and transposase and it also contains the guide RNA gene. This operon was set under the control of the lactococcal promoter P5 [23]. The donor plasmid pL2Donor was created on the base of pTRKL2 vector and it contained the chloramphenicol resistance gene which was flanked by the transposon shoulders [24].

The transformation of the *L. lactis IL1403* by these plasmids (the cr RNA sequence targeting the beta-galactosidase gene was used) yielded no detectable insertion of the marker [25]. The plasmids containing the non-targeting crRNA were used as a negative control, and they did not provide any transposition. We suggested that the lack of the transposition was due to insufficient activity of the ribosome binding sites (RBS) from *V. cholerae* in the *L. lactis* cells. To improve the translation of the proteins, we replaced all of the RBS by the universal RBS AGGAGG in a pL2INT plasmid, and we obtained a plasmid pLcINT.

We also noticed a background chloramphenicol resistance in the pL2Donor-containing cells because of the leakage of the transcription from the vector promoter. In order to tackle this problem, we introduced the bilaterally active transcription terminating sites on both sides of the integrative sequence to generate the plasmid pLcDonorT which did not exhibit any leakage phenotypes.

The modified system allowed for the editing (knockout) of the *L. lactis* beta-galactosidase gene with an efficiency of about 7 CFU per 10^8^ of the cells.

### 2.2. Promoter Optimization

To improve the editing efficiency, we tried to alter the transcription rate of the effector operon by swapping the promoters. To do this, we created the pLcINT plasmid variants in which the constitutive promoter P5 was replaced by the promoters P4, P6, P7 or P8 [26] without altering of the gene order or other elements of the vector design. We co-transformed *L. lactis IL1403* by each of these vectors in combination with the same pLcDonor plasmid and analyzed the editing efficacy.

**Figure 1:**
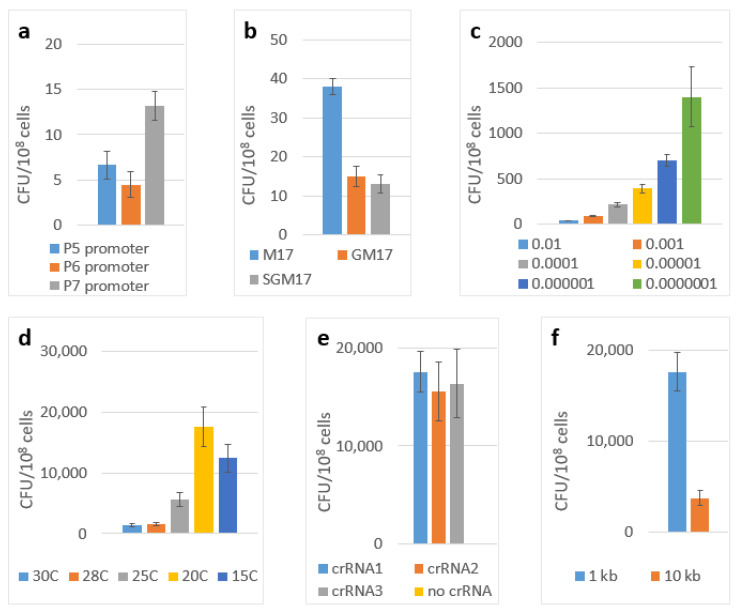
Transposition efficiencies. The number of chloramphenicol-resistant (cm) CFUs per 10^8^ cells of *L. lactis IL1403* which were transformed by the two-plasmid genome-editing system depending on: (**a**) transcription promoter used; (**b**) the carbon sources of solid medium; (**c**) the inoculum size; (**d**) the incubation temperatures; (**e**) the guide RNA choice; (**f**) the total size of the integrating fragment. The error bars indicate the standard deviation values from three biological replicates.

The construct with the P4 promoter yielded no editing at all, which was probably because of its weaker transcription rate when it was compared to that of the other promoter variants. The P8-containing construct significantly suppressed the growth of the cells, which may be explained by it having an overly strong transcription rate. The efficiency of the editing with the promoters P5, P6 and P7 were 7, 4 and 13 CFU per 10^8^ of the cells, respectively (Figure 1a).

We then attempted to further improve the system’s efficiency by increasing the crRNA synthesis. To achieve this result, we created the versions of the pLcINT plasmid in which the operon encoding all of the necessary proteins were put under the control of the P7 promoter, and the crRNA gene was placed downstream of it under the control of the promoters P5, P6 or P8. However, all of these versions showed no editing activity.

**Figure 2:**
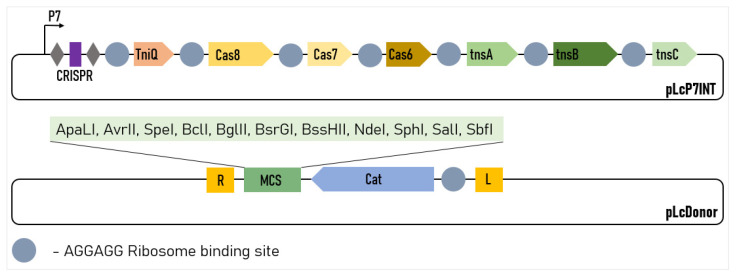
Design of the two-plasmid *L. lactis* genome-editing system. The CRISPR array comprises two repeats (grey diamonds) and a single spacer (purple rectangle). R and L represent the left and right transposon shoulders, respectively.

So, the most efficient version of the system contained the pLcINT plasmid with the promoter P7 and the modified RBS of all of the protein genes. This plasmid was named pLcP7INT (Figure 2).

### 2.3. Optimization of the Experiment Conditions

To further improve the yield of the integrant clones, we optimized the condition of the cultivation of the transformants of *L. lactis IL1403* carrying the developed two-plasmid editing system (pLcP7INT—pLcDonor system). Based on the published results [24], we suggested that extending the period of the active growth by lowering the cultivation temperature and the size of the inoculum may increase the outcome of the positive clones. We optimized these parameters, and we also investigated the effect of the carbon source type that was used in the cultivation medium.

Initially, we performed our experiments using the SGM17 medium (containing lactose, glucose and sucrose) that was inoculated by 10^−2^ volume of the overnight plasmids-containing culture. The incubation was performed at 30 °C. First, we chose the optimal carbon source. We compared the standard M17 medium containing lactose only with the variants in which lactose was supplemented by glucose, sucrose or a combination of glucose and sucrose. The results of these experiments are shown in Figure 1b. The best results were obtained with the M17 medium (lactose only).

**Figure 3:**
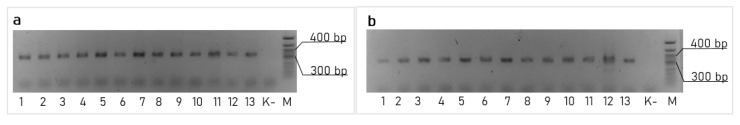
PCR confirmation of the target edition: (**a**) transposition of the 1 kbp insert, performed by the pLcDonor and pLcINT plasmids pair with a crRNA01; (**b**) transposition of the 10 kbp insert performed using the pLcDonor_10 kb and pLcINT plasmids pair with a crRNA01.

Then, we optimized the inoculum size by comparing 10^−2^, 10^−3^, 10^−4^, 10^−5^, 10^−6^ and 10^−7^ of the volume of the overnight cultures using the M17 medium and by incubating them at 30 °C (Figure 1c). The best results were obtained with the inoculum of 10^−6^. Finally, we tested these conditions (the M17 medium with the inoculum size of 10^−6^) in combination with the incubation temperatures: 30 °C, 28 °C, 25 °C, 20 °C and 15 °C. The results of these experiments are shown in Figure 1d. By combining the best conditions from all of these experiments (the medium M17, an inoculum size of 10^−6^ and the incubation at 20 °C), we were able to improve the editing efficiency up to 17,600 CFU per 10^8^ cells (~2 × 10^−4^). Successful editing was confirmed by the PCR screening of the clones with the primers matching the insertion and the flanking genome region with the subsequent Sanger sequencing of the PCR product (Figure 3a).

### 2.4. Variation of Guide RNA Sequences

The influence of the guide RNA choice on the editing efficiency of the Cas9-based systems has been reported previously [27]. Previous reports have demonstrated that several factors may affect the crRNA targeting efficiency, including the alignment of spacer sequences to the genome, Cas9 guide sequence, and the target site of crRNA [28,29,30].

To test the influence of this factor under our conditions we designed three different guide RNA sequences targeting different sites in the beta-galactosidase gene proceeded by the 5′-CC protospacer adjacent motif. The resulting efficiency of the integration of 1 kbp cassette showed no significant difference between these variants (Figure 1e).

### 2.5. Integration of Large Insertions

The possibility of incorporating large genetic cassettes into the *L. lactis* genome, potentially enabling the easy incorporation of the new metabolic pathways, anti-phage systems or large heterogeneous antigens into this organism was of particular interest for us. To demonstrate the possibility for us to integrate large constructs, we modified the pLcDonorT vector incorporating the multiple cloning sites (MCS) fragment downstream of the chloramphenicol marker (Figure 2) to obtain the pLcDonor plasmid. Using the ApaLI and BssHII sites of the MCS, we cloned the 9 613 bp insert into the pLcDonor vector, thus increasing the total size of the integrating fragment up to 10 350 b.p. The resulting pLcDonor_10 kb plasmid was co-transformed into the *L. lactis* cells with the pLcI7INT plasmid, containing a crRNA1, which targets a genomic site in the beta-galactosidase gene with CC 5′-PAM, and the transformants were processed under the above-mentioned optimal conditions. The efficiency of the integration of the long cassette into the beta-galactosidase gene was 3760 CFU per 10^8^ (~4 × 10^−5^) of the cells of the culture, which was only about 5 times lower than which was achieved with the 1 kBp-long integrative sequence (Figure 1f). The editing was confirmed by PCR screening and Sanger sequencing as it was performed for the 1 kbp insertion (Figure 3b).

## 3. Discussion

*L. lactis* is an important industrial microorganism, and also, it is widely used as a model object for LAB biology research. The lack of efficient tools for the manipulation of the *L. lactis* genome represents, therefore, a significant practical problem. Although the suggested systems based on RecA or RecT-mediated recombination, including the systems employing Cas9 to enhance the recombination in the target locus [8,31], allow for a high efficiency of the gene knock-out process by short (ca. 150 b.p.) insertions, the low efficiency of the recombination with a longer size of the non-homologous region in the integrative sequence restricts their usage for the incorporation of bigger fragments.

The system that has been developed by us has substantial advantages when it is compared to the previously reported approaches based on RecA-mediated homologous recombination. It does not require there to be large shoulders of the homologous sequences, thereby reducing the genetic engineering efforts to the cloning of a small synthetic crRNA gene and the preparation of the custom insertion cassette (if this is necessary). The system has also been confirmed to integrate longer DNA fragments (10 kbp) with an efficiency of 4 × 10^−5^, which is only slightly (5 times) lower than the integration rate that was achieved with the 1 kbp fragment. This result allows us to suggest that the fragments significantly exceeding 10 kbp in size can be successfully incorporated into the LAB genomes with our system. However, the most important advantage is the independence of the transformation efficacy that can be obtained in a particular LAB strain. A single transformant colony containing the two plasmids of our system is sufficient to grow the culture and achieve the targeted fragment incorporation. The number of the edited clones that are obtained is also higher when it is compared to the results that have been reported in the literature (4 × 10^3^ for 10 kbp insertion vs. 10^2^ for 5 kbp insertion [10]). This may be important if a library of diverged sequences is created using the editing system, though the independence of the clones has to be tested for each particular experimental conditions (the bacterial strain and the constructs that are used).

The system that is based on the guide RNA-directed transposition which has been developed by us creates an avenue for the easy modification of the LAB genomes, incorporating additional genetic material in their chromosomes. This approach may be instrumental for creating efficient diacetyl or butanedione producing strains [32,33], and also, it may be helpful in solving the problem of bacteriophage lysis in the industry through the incorporation of new anti-phage systems, the known diversity of which has recently widely expanded [34].

It is noteworthy to mention that, initially, the very low efficiency of the editing with our system could be strongly improved by the optimization of both the vector design (correct choice of the RBS and promoter) and of the experiment conditions, among which the size of the inoculum and the incubation temperature showed the most significant influence. Interestingly, the increase in the editing efficiency that was achieved with our system under a lower incubation temperature (the temperature of 15 °C significantly hindered the culture growth, and so, 20 °C appears to be the lowest operational value) correlates with the results of the study by Phuc Leo H. Vo et al. in 2020 where the improvement of the transposition in *E. coli* has been observed at a lower temperature.

We also speculate that the most active transposition takes place during the exponential culture growth phase, and it is strongly decreased during the transition to the stationary phase. This may explain the improvement of the editing efficiency with the decrease of the inoculum size.

The limitation of our system is the lack of the possibility to conduct direct gene replacement or the deletion of the genetic material. However, if the replacement of essential genes is required, the possibility to, first, incorporate the new alleles, and then, delete the unwanted material using an existing tool for short fragments incorporation with the subsequent use of site-specific recombination-based tools could make the task potentially feasible.

## 4. Materials and Methods

### 4.1. Bacterial Strains and Their Cultivation

All of the bacterial strains that were used in this study are listed in Table S1. The bacterial strain *L. lactis IL1403* and its derivatives were grown at 30 °C in M17 broth (Merck) supplemented with 0.5% glucose (GM17) without agitation unless other conditions are specified. For the solid media, agar (1.0% *w*/*v*) was added to the GM17 broth. For the transformation, the electrocompetent cells were prepared as described by M. Papagianni et al. [35]. The antibiotics were added to the GM17 medium when they were required at the following concentrations: 5 μg/mL for chloramphenicol or erythromycin and 150 μg/mL for spectinomycin. The *E. coli* strains were grown in LB medium and incubated at 37 °C while under agitation. Chemically competent *E. coli NEB10b* cells (New England Biolabs) were used for the cloning. When they were needed, chloramphenicol, erythromycin and spectinomycin were added to a final concentration of 20 μg/mL, 150 μg/mL and 50 μg/mL, respectively, in the LB. For the solid media, the agar (1.5% *w*/*v*) was added to the LB.

### 4.2. Reagents and Enzymes

The plasmids were purified from overnight bacterial cultures using a Monarch Plasmid Miniprep Kit (New England Biolabs). The chromosomal DNA was purified from overnight bacterial cultures using a Monarch Genomic Miniprep Kit (New England Biolabs). Prior to the chromosomal DNA extraction, the *L. lactis IL1403* cultures were treated with lysozyme (30 mg/mL, 30 min, 37 °C). The restriction enzymes were purchased from New England Biolabs. The polymerase chain reactions (PCR) were performed with Taq polymerase (New England Biolabs) for screening purposes and with Q5 high-fidelity DNA polymerase (New England Biolabs) for cloning purposes. The oligonucleotides were purchased from Eurogen. All of the primers and oligonucleotides that were used in this study are listed in Table S2.

### 4.3. Plasmid Construction

All of the plasmids that were used in this study are listed in Table S1. The Supplementary Materials contain the plasmids sequences in a FASTA format in the “FASTA Plasmids 007” file. All of the crRNAs that were used in this study are listed in Table S3. The transcription terminators synthesis was ordered from GenScript Company (Rijswijk, Netherlands). The CRISPR array and the genes TniQ, Cas8, Cas7 and Cas6 were amplified from the pQCascade plasmid. The genes and TnsA, TnsB and TnsC were amplified from the pTnsABC. The obtained fragments were cloned into a pTRKL2Sp vector, yielding pL2INT. To replace the native ribosome binding site of each gene, we introduced the required sequence by PCR mutagenesis and cloned the fragments back into the pL2INT plasmid, yielding the pLcINT vector. To generate the pL2Donor plasmid, the transposon shoulders were amplified from the pDonor vector and the chloramphenicol resistance gene with the necessary ribosome-binding site (but without promoter) was amplified from pTRK669 [36]. The fragment encoding the transcription terminators and MCS were synthesized by GenScript. Further derivatives of these plasmids were created using a combination of methods: restriction digestion ligation, the ligation of the hybridized oligonucleotides and an around-the-horn PCR. The plasmids were cloned and propagated in the NEB10b cells (New England Biolabs) and verified by a Sanger sequencing (Evrogen, Moscow, Russia).

### 4.4. Transposition Experiments

All of the transposition experiments were performed in the *L. lactis IL1403* cells. For the experiments, the plasmids pLcDonor, pLcINT or their derivatives were electroporated into the cells sequentially: first, the pLcDonor plasmid was electroporated, and then, the pLcINT or its derivatives were electroporated. The transformants colonies containing both of the plasmids were transferred into 5 mL of the liquid GM17 medium supplemented with 5 μg mL^−1^ of erythromycin and 150 μg mL^−1^ of spectinomycin, and they were incubated overnight at 30 °C without being agitated. The cultures, OD600 nm, were measured and adjusted to the value of 1.5 in order to standardize the inoculum in all of the experiments. The flasks containing 5 mL of the fresh chosen liquid medium (see the Results Section 2.3), containing the erythromycin and spectinomycin in the above specified concentrations, were inoculated by a chosen volume of the inoculum (see the Results Section 2.3), and they were grown without being agitated up to the maximal OD600 nm, which took 24–72 depending on the incubation temperature (see the Results Section 2.3). The cultures that were obtained were plated on the plates with the solid GM17 medium containing 5 μg mL^−1^ of chloramphenicol. After 24–48 h of incubation at the chosen temperature (see the Results Section 2.3), we obtained the colonies of the modified (edited) bacteria. The total cell count of the culture was simultaneously determined by the plating of an appropriate dilution on the GM17 plates without an antibiotic. The integration efficiency was calculated as a ratio of the integrants CFU which were determined by the chloramphenicol selection to the total CFU counts. The cultures containing both of the plasmids but without any crRNA gene having been inserted into the pLcINT (or its derivatives) were used as the negative controls.

### 4.5. PCR and Sanger Sequencing Analysis of Transposition Products

The overnight cultures of the chloramphenicol-resistant clones were grown in the liquid GM17 medium without antibiotics, and the chromosomal DNA was extracted. One microgram of this DNA was used for the PCR analysis with Q5 Hot Start High-Fidelity DNA Polymerase (New England Biolabs) per 25 μL reaction volume. The reactions were generally subjected to 36 thermal cycles with an annealing temperature of 58 °C. The primer pairs which contained one genome-specific primer and one transposon-specific primer were used. The sequences of the oligonucleotides that were used in this study available in a supplementary materials (Table S2). The negative control samples were always run in parallel with the experimental samples to identify the possible non-specific amplification. To map the integration, the bands of the PCR fragments were gel purified using the Monarch DNA Gel Extraction Kit (New England Biolabs), and they were submitted for commercial Sanger sequencing to the Evrogen company.

## 5. Conclusions

The guide RNA-directed transposition-based system for *L. lactis* genome editing makes it possible to easily incorporate large fragments of foreign DNA into the chromosomes of LAB, thus creating an avenue for efficient metabolic engineering, anti-phage protection and other modifications of these organisms. The convenient design of the system and the usage of the RBS and promoters sequences that are recognizable by many other bacteria makes the tool potentially useful, directly or after some moderate adaptation efforts, for the work with other groups of bacteria such as *Enterobacteriaceae*.

## Data Availability

Data are contained within the article or the Supplementary Material.

## Supplementary Materials

The following supporting information can be downloaded at: https://www.mdpi.com/article/10.3390/ijms232213978/s1.
