# Supplementary material for: New Effective Method of Lactococcus Genome Editing Using Guide RNA-Directed Transposition
Source: Int J Mol Sci. 2022 Nov 12;23(22):13978. doi: 10.3390/ijms232213978 (PMC9696066; doi:10.3390/ijms232213978)
Supplement: Supplementary file 1 [file ijms-23-13978-s001.zip › Table S1-3.pdf]

Table S1 | Bacterial strains and plasmids used in this study.

Strains	Relevant informatiom	Reference or source
E. coli NEB10b	Competent cells, cloning	New England Biolabs
L. lactis IL1403	*L. lactis* plasmid free	[25]
Plasmids		
pTRKL2	Low copy number vector, Em^r;	[24]
pTRKL2Sp	pTRKL2 derivative, Sp^r;	This study
pTRK669	Cm^r ;	[36]
pQCascade	*E. coli* transposon effector plasmid, *tniQ-cas8-cas7-cas6,* Sp^r;	[22]
pTnsABC	*E. coli* transposon effector plasmid, t*nsA-tnsB-tnsC,* Kan^r;	[22]
pDonor	*E. coli* mini-transposon donor, Amp^r;	[22]
pL2INT	pTRKL2Sp derivative, P_5-*tniQ-cas8-cas7-cas6-tnsA-tnsB-tnsC.* native RBSs. Sp^r	This study
pL2INT_crRNA1	pL2INT derivative, P_5-*tniQ-cas8-cas7-cas6-tnsA-tnsB-tnsC.* native RBSs, crRNA1, $Sp^{r;}$	This study
pL2Donor	*L. lactis* mini-transposon, Cm-resistance gene under AGGAGG RBS without promoter, Em^r;	This study
pLcINT	pL2INT derivative, P_5-*tniQ-cas8-cas7-cas6-tnsA-tnsB-tnsC, AGGAGG* RBSs, Sp^r;	This study
pLcDonorT	*L. lactis* mini-transposon, Cm-resistance gene under AGGAGG RBS without promoter, terminators from upstream from right transposition end, Em^r ;	This study
pLcDonor	pLcDonorT derivative, contain MCS, Em^r ;	This study
pLcP4INT	pLcINT derivative, P_4-*tniQ-cas8-cas7-cas6-tnsA-tnsB-tnsC, AGGAGG* RBSs, Sp^r;	This study
pLcP6INT	pLcINT derivative, P_6-*tniQ-cas8-cas7-cas6-tnsA-tnsB-tnsC, AGGAGG* RBSs, Sp^r;	This study
pLcP7INT	pLcINT derivative, P_7-*tniQ-cas8-cas7-cas6-tnsA-tnsB-tnsC, AGGAGG* RBSs, Sp^r;	This study
pLcP8INT	pLcINT derivative, P_8-*tniQ-cas8-cas7-cas6-tnsA-tnsB-tnsC, AGGAGG* RBSs, Sp^r;	This study
pLcINT_crRNA1	pL2INT derivative, P_5-*tniQ-cas8-cas7-cas6-tnsA-tnsB-tnsC, AGGAGG* RBSs, crRNA1, Sp^r;	This study
pLcP4INT_crRNA1	pLcP4INT derivative, P_4-*tniQ-cas8-cas7-cas6-tnsA-tnsB-tnsC, AGGAGG* RBSs, crRNA1, Sp^r;	This study
pLcP6INT_crRNA1	pLcP6INT derivative, P_6-*tniQ-cas8-cas7-cas6-tnsA-tnsB-tnsC, AGGAGG* RBSs, crRNA1, Sp^r;	This study
pLcP7INT_crRNA1	pLcP7INT derivative, P_7-*tniQ-cas8-cas7-cas6-tnsA-tnsB-tnsC, AGGAGG* RBSs, crRNA1, Sp^r;	This study
pLcP8INT_crRNA1	pLcP8INT derivative, P_8-*tniQ-cas8-cas7-cas6-*	This study

	tnsA-*tnsB*-*tnsC, AGGAGG* RBSs, crRNA1, Sp^r;	
pLcP7INT_crRNA2	pLcP7INT derivative, P$_7$-*tniQ*-*cas8*-*cas7*-*cas6*-*tnsA*-*tnsB*-*tnsC, AGGAGG* RBSs, crRNA2, Sp^r;	This study
pLcP7INT_crRNA3	pLcP7INT derivative, P$_7$-*tniQ*-*cas8*-*cas7*-*cas6*-*tnsA*-*tnsB*-*tnsC, AGGAGG* RBSs, crRNA3, Sp^r;	This study
pLcDonor_10kb	pLcDonor derivative. Contains 9 613 bp insert by NotI, BssHII, Em^r ;	This study

Em^r – erythromycin resistance, Cm^r – chloramphenicon resistance, Sp^r – spectinomycin resistance Amp^r – ampicillin resistance, Kn^r – kanamycin resistnace

Table S2 | Description and sequence of oligonucleotides used in this study.

Oligonucleotid description	Oligonucleotid DNA sequence (5'-3')	Target	Function
#001	ctctcgaggaaaaagaaaatgtttttgtatttttagaatccc ttttctataaatcaattctaattataaggacctgatgattg agtgataatgctagtttgaagcattcttagtaagaaa	P5	Assembly of pL2INT
#002	gctagtttgaagcattcttagtaagaaagtgatttttttata aatggtttatagaataaattgtacagcgtttaattggact tgctctctgaaataacgtaaaattgtagtgaggaggacg	P5	
#003	atatagcgcgcgaaaaagaaaatgtttttgtatttttagaa tcc	P5	
#004	cgtttaattggacttgctctctgaaataacgtcaattcccctgt agaaataattttg	P5	
#005	caaaattatttctacaggggaattgacgttatttcagagagcaag tccaattaaacg	P5	
#006	gcataatcgaaattaatacgactcac	CRISPR	
#007	ttgtacacggccgcataatc	CRISPR	
#008	caattcccctgtagaaataattttg	CRISPR	
#009	atatacctaggtcagattaagggtacaggctgaaacg	Cas6	
#010	cctaaaccttacagcgatgaaagt	TniQ	
#011	ggacctgatgattgagtgataatgc	P5	
#012	gcattatcactcaatcatcaggtcc	P5	
#013	atatagcgcgccgttttacaacgtcgtgactgg	pTRKL2Sp	
#014	atatagtcgaccttccgtcacaggtatttattcg	pTRKL2Sp	
#015	ccttccgtcacaggtatttattcgg	pTRKL2Sp	
#016	ccagtcacgacgttgtaaaacg	pTRKL2Sp	
#017	atataactagtctttaataaggagatataccatggcgac	TnsA	
#018	agaggcgagcaatttgacc	Cas6	
#019	gctcgcaatctaacgaaatctc	TnsA	

#020	atatagtcgacttattcgaaaaagtttttatttctacctgg	TnsC	
#021	atatagcggccgctgttgatgcaaccataaagtgatattt aataattatttataatc	LT end	Assembly of pL2Donor
#022	aattactgcagtaagttataaaagccagtcattaggccta	CmR F	
#023	atattggatcctggaggaagaaagattatgaactttaataaaatt gatttagacaattgg	CmR R	
#024	atataggatccgacgtcattagctgttgatacaaccataaa atgataattacacc	RT end	
#025	atatagcggccgccgttttacaacgtcgtgactgg	pTRKL2	
#026	atataggatccttccgtcacaggtatttattcgg	pTRKL2	
#027	acaggtacatcattctgtttgtgatgg	CmR R	
#028	ggaaacaatttccccgaacc	CmR R	
#029	aattactgcagtaagttataaaagccagtcattaggccta	CmR F	
#030	atattggatcctggaggaagaaagattatgaactttaataaa attgatttagacaattgg	CmR R	
#031	ccagtcacgacgttgtaaaacg	pTRKL2	
#032	gacgtcattagctgttgatacaaccataaaatgataa ttacacc	RT end	Assembly of pLcDonorT
#033	atatagacgtctgcgtgactggcaagagatcc	Terminators F	
#034	atataggatccggaaatattcattctaattggcaagagc	Terminators R	
#035	gcatcagctcgattctcctcctc	Terminators	
#036	gtaagtgcagtaaggtgcacatcgcctaggactagtaagca tgatcaagatcttgtacaactttcatcgctgtaaggtttagggcg cgcatatgcatgccagtgtcgaccctgcagggtgcaataa cttcgtatagcatacattatacgaacggtat	MCS	Assembly of pLcDonor
#037	ctagataccgttcgtataatgtatgctatacgaagttattgcaccctgc agggtcgacactggcatgcatatgcgcgccctaaaccttacagcg atgaaagttgtacaagatcttgatcatgcttactagtcctagg cgatgtgcaccttactgcacttactgca	MCS	
#038	tttccggatccttccgtcacaggtatttattcgg	pTRKL2	
#039	gttgtttgtcggtgaacgc	Terminators	
#040	gcgttcaccgacaaacaac	Terminators	
#041	ccagtcacgacgttgtaaaacg	pTRKL2	

#042	actttcatcgctgtaaggtttagg	MCS	
#043	cctaaaccttacagcgatgaaagt	MCS	
#044	atataggatccaattacatattttatgtttggaggaag aaagattatgtttttgcaaagacctaaaccttacagc	LcRBS TniQ F	Assembly of pLcINT
#045	aaaaaagcttcctaggttaccacctcgacacataaaacg	TniQ R	
#046	aaaaaaactagtggaggaagaaagattatgcaaactctg aaagaactaatcg	LcRBS Cas8 F	
#047	tttctgcagtcataggctagatttgtcggtagtttc	LcRBS Cas8 R	
#048	aaactgcagtggaggaagaaagattatgaaactaccgac aaatctagccta	LcRBS Cas7 F	
#049	tttttgtcgacttttctagatcagtctcctttatgctggaa catcc	LcRBS Cas7 R	
#050	tttactagtggaggaagaaagattatgctgactgtgaaa tggtattataagacaatcacc	LcRBS Cas6 F	
#051	aaacctaggtcagattaagggtacaggctgaaacg	LcRBS Cas6 R	
#052	aaaactagtggaggaagaaagattatggcgaca agtttacctacg	LcRBS TnsA F	
#053	aaaaagcttagccataactggccaactca	LcRBS TnsA R	
#054	aaaaagcttggaggaagaaagattatggctaagaaa gggttctcaag	LcRBS TnsB F	
#055	aaacctaggtcagacataatcaatatcccaatcg	LcRBS TnsB R	
#056	aaaactagtggaggaagaaagattatgagtgaaac gcgtgaggct	LcRBS TnsC F	
#057	tttgtcgacttattcgaaaaagtttttatttctacctgg	LcRBS TnsC R	
#058	atatagtcgaccttccgtcacaggtatttattcgg	pTRKL2Sp	
#059	cgatcagattgcatcaatgg	TniQ	
#060	cctgctcgatcgagccaaat	Cas8	
#061	tcggttaccacttgttagaagagc	Cas8	
#062	cctatgatgtctcaggtcaacca	Cas7	
#063	gaagccactgagccactaagg	Cas7	
#064	ccagagttgtgcaacaacga	Cas6	
#065	agaggcgagcaatttgacc	Cas6	
#066	gctcgcaatctaacgaaatctc	TnsA	
#067	ccaatctgcaagatgcttgg	TnsA	
#068	gcatttcccgaaacaattgc	TnsB	
#069	aaattgaagaaatcgcagatcg	TnsB	
#070	cctacatgcatgatggtctatgg	TnsC	
#071	acgataaccgaagaagatttcg	TnsC	

#072	atatagcgcgcgaaaaagaaaatgtttttgtatttttagaa tccctttttctataaatcaattctaattataaggacctgatg attgagtgataatgctagtttgaagcattcttagtaagaaa	P4	Assembly of pLcP4INT
#073	actagtcctaggacgttatttcagagagcaagtccaatta aacgctgtacaatttattctataaaccatttataaaaaatc actttcttactaagaatgcttcaaactagcattatcactcaa tcatcagg	P4	
#074	tggacttgctctctgaaataacgtcctaggactagtcaattcc cctgtagaaataattttg	P4	
#075	caaaattatttctacaggggaattgactagtcctaggacgttattt cagagagcaagtcca	P4	
#076	ggacctgatgattgagtgataatgc	P4	
#077	gcattatcactcaatcatcaggtcc	P4	
#078	atatagcgcgcagccattttttgcaaaaaaagcatattagta actaacaaaaatataaacaaaagtaaaataatagaattta atgtacgaaatttattacaaatttgtattatattatgctata	P6	Assembly of pLcP6INT
#079	atatagcgcgcagccattttttgcaaaaaaagcatattagtaa ctaacaaaaatataaacaaaagtaaaataatagaatttaatgt acgaaatttattacaaatttgtattatattatgctata	P6	
#080	catctcttgcaaaaatgatttaaaatcaaacaattcccct gtagaaataattttg	P6	
#081	caaaattatttctacaggggaattgtttgattttaaatcatttt gcaagagatg	P6	
#082	gaaaatgtaatcaataaaatacatctcttgc	P6	
#083	gcaagagatgtattttattgattacattttc	P6	
#084	atatatagcgcgcagtgattattataaaagacaattctgataa agagttgtttttttaatttcttaagcaaatcttattcttgataag caaactatttttgtgctataataaaaatatc	P7	Assembly of pLcP7INT
#085	gcaaactatttttgtgctataataaaaatatcttcagggcac cgtgtaattcgggaccggcggtaaatagggctttgacctt atgactccgcgattcgctacggcgattgaagcagtgag	P7	
#086	gctacggcgattgaagcagtgagaatctgctagcgacagt aaagtctggatggaagaagatgaacaatttttgtagtgtaa tcaacttcggtggattactgtttttagccaaaccaaaa	P7	
#087	gattactgtttttagccaaaccaaaaatgtctatcaacttcc tcgaaatttcaattcc	P7	
#088	ctatcaacttcctcgaaatttcaattcccctgtagaaataattttg	P7	
#089	ctatcaacttcctcgaaatttcaattcccctgtagaaataattttg	P7	

#090	ggtaaatagggctttgaccttatgac	P7	
#091	gtcataaggtcaaagccctatttacc	P7	
#092	atatagcgcgcgataaaatttctaatgattttttaggacaat tatttctcataaaaagcagatttagaaagaaaattgtatt tttttaacagctttgactgcccttttggaagagtttat	P8	Assembly of pLcP8INT
#093	ccatttctgctgctatatcaatagcaaaactaactaattcta ttatacataaactcttccaaaaagggcagtcaaagc	P8	
#094	gctattgatatagcagcagaaatggcaattcccctgtagaaataattttg	P8	
#095	caaaattatttctacaggggaattgccatttctgctgctatatcaatagc	P8	
#096	gctttgactgcccttttgg	P8	
#097	ccaaaaagggcagtcaaagc	P8	
#098	atatagtcgacgaaaaagaaaatgtttttgtatttttagaatcc	P5	Assembly of plasmids with a separate crRNA expression.
#099	atataactagtaaacataaaatatgtaatttgg	Terminator R	
#100	atatagtcgacgataaaatttctaatgattttttaggac	P8	
#101	atatagtcgacagccatttttttgcaaaaaaagc	P6	
#102	atatagtgcacttatttggcattcaaagctgcaac	10kb insert	Assembly of pLcDonor_10kb
#103	ggtttgtaccgataactacgattttcattatatctctcca tttctgctgctatatcaatagc	10kb insert	
#104	gctattgatatagcagcagaaatggagagatataatg aaaatcgtagttatcggtacaaacc	10kb insert	
#105	atgaaaatcgtagttatcggtacaaacc	10kb insert	
#106	atatagcggccgctagcagaaggaaggaattg	10kb insert	
#107	ctcacaggaacaaccaaacaacgtgtcgacttatt	10kb insert	
#108	tttgtcgacttattcttcaacttcaaattctgg	10kb insert	
#109	atgactaaacaaacttttgatgatttaattaatc	10kb insert	
#110	gattaattaaatcatcaaaagtttgtttagtcatgtaatcact ccttcttaattacaaatttttag	10kb insert	
#111	ctaaaaatttgtaattaagaaggagtgattacatgactaaac aaacttttgatgatttaattaatc	10kb insert	
#112	ctaaaaatttgtaattaagaaggagtgattac	10kb insert	
#113	catatgcgcgcggttcgtgttcgtgctgacttg	10kb insert	
#114	aacagcaaagaatggcgg	10kb insert	
#115	gattcaggaacttctgacaataacc	10kb insert	
#116	gggattagttgggttatcgacc	10kb insert	

#117	gcaaataacgatatgataaaatcaagg	10kb insert	
#118	gtgcgctcaataaggtttgg	10kb insert	
#119	cctagataactcatgttgctattacg	10kb insert	
#120	gctttgcttgatttattcttcctcc	10kb insert	
#121	tgaaccgccagagctacttgg		Primers for screening and sequencing
#122	atcgaagatgaagtagagttcaacc	pL2INT	
#123	ccgcatattgcaattgatgg	pL2INT	
#124	ttcactctctcctgttgcatcg	pL2INT	
#125	tccatcacttacagctttctttgg	pL2INT	
#126	atcacctttagctcagagactgatcc	pL2INT	
#127	ttgtacttacaccttggccttgg	pL2INT	
#128	cataaaggagactgactgtgaaatgg	pL2INT	
#129	ggaactcgatttttagtagctatggctt	pL2INT	
#130	cgttatgcttcgaaagatgagc	pL2INT	
#131	ccattagcttagcacctaatatcaagg	pL2INT	
#132	ccagacctactcgtaactgataatgg	pL2INT	
#133	agttatttgggtgttggatgagg	pL2INT	
#134	ttcgggtgtaggtaaaacgacc	pL2INT	
#135	agccctaccttttgaaaaacagg	pL2INT	
#136	ccgaataaatacctgtgacgg	pL2INT	
#137	gcgagaaaatggctacgcct	TniQ-F	
#138	gtacacgctgcctcatgacc	TniQ-R	
#139	atttcgtccactcacaccgc	Cas8-F	
#140	tcggtaattcacccagggct	Cas8-R	
#141	accttgcacttgccaacgaa	Cas7-F	
#142	tccgacccttagtggctcag	Cas7-R	
#143	gaggcgagcaatttgaccca	Cas6-F	
#144	agggtacaggctgaaacgagt	Cas6-R	
#145	acgccctcagcaattacgac	TnsA-F	
#146	ggttgagcgcaaaagcgaac	TnsA-R	
#147	tgtgtcggtttcccttgcac	TnsB-F	
#148	gttgtcgggcgtctcaactt	TnsB-R	
#149	aattaatccctgcggtcggc	TnsC-F	
#150	taaaggggcgcagttcaacc	TnsC-R	
#151	agatgagttcatctgaaaggaatgg	L. lactis IL1403 beta-D-galactosidase	
#152	gttacttccataacctgactcattcc	L. lactis IL1403 beta-D-galactosidase	

Table S3 | crRNA used in this study.

crRNA description	(5'-3') crRNA sequence ; PAM: 5'-CC-3'	target
crRNA1	AGTAGTTTCAAATTGGAATAGGTTGCCAATGC	Beta-
crRNA2	TATGGACTTGTTAGAAAAGCAATCACTGAATG	galactosidase of
crRNA3	TAAAAATTGGTTAGAACTGACGGAGAGTAAAA	*L.lactis IL1403*
